# Conventional and Zero Tillage with Residue Management in Rice–Wheat System in the Indo-Gangetic Plains: Impact on Thermal Sensitivity of Soil Organic Carbon Respiration and Enzyme Activity

**DOI:** 10.3390/ijerph20010810

**Published:** 2023-01-01

**Authors:** Asik Dutta, Ranjan Bhattacharyya, Raimundo Jiménez-Ballesta, Abir Dey, Namita Das Saha, Sarvendra Kumar, Chaitanya Prasad Nath, Ved Prakash, Surendra Singh Jatav, Abhik Patra

**Affiliations:** 1 Division of Soil Science and Agricultural Chemistry, ICAR-Indian Agricultural Research Institute, New Delhi 110 012, India; 2 ICAR—Indian Institute of Pulses Research, Kanpur 208 024, India; 3 Centre for Environment Science and Climate Resilient Agriculture, ICAR-Indian Agricultural Research Institute, New Delhi 110 012, India; 4 Department of Geology and Geochemistry, Autónoma University of Madrid, 28049 Madrid, Spain; 5 ICAR- Indian Institute of Farming Systems Research, Modipuram 250 110, India; 6 Department of Soil Science and Agricultural Chemistry, Institute of Agricultural Sciences, Banaras Hindu University, Varanasi 221 005, India; 7 Krishi Vigyan Kendra, Narkatiaganj, West Champaran 845 455, India

**Keywords:** activation energy, aggregate-associated carbon, carbon mineralization, glomalin, temperature sensitivity of SOC decomposition (Q_10_)

## Abstract

The impact of global warming on soil carbon (C) mineralization from bulk and aggregated soil in conservation agriculture (CA) is noteworthy to predict the future of C cycle. Therefore, sensitivity of soil C mineralization to temperature was studied from 18 years of a CA experiment under rice–wheat cropping system in the Indo-Gangetic Plains (IGP). The experiment comprised of three tillage systems: zero tillage (ZT), conventional tillage (CT), and strip tillage (ST), each with three levels of residue management: residue removal (NR), residue burning (RB), and residue retention (R). Cumulative carbon mineralization (C_t_) in the 0–5 cm soil depth was significantly higher in CT with added residues (CT-R) and ZT with added residues (ZT-R) compared with the CT without residues (CT-NR). It resulted in higher CO_2_ evolution in CT-R and ZT-R. The plots, having crop residue in both CT and ZT system, had higher (*p* < 0.05) Van’t-Hoff factor (Q_10_) and activation energy (Ea) than the residue burning. Notably, micro-aggregates had significantly higher Ea than bulk soil (~14%) and macro-aggregates (~40%). Aggregate-associated C content was higher in ZT compared with CT (*p* < 0.05). Conventional tillage with residue burning had a reduced glomalin content and β-D-glucosidase activity than that of ZT-R. The ZT-R improved the aggregate-associated C that could sustain the soil biological diversity in the long-run possibly due to higher physical, chemical, and matrix-mediated protection of SOC. Thus, it is advisable to maintain the crop residues on the soil surface in ZT condition (~CA) to cut back on valuable C from soils under IGP and similar agro-ecologies.

## 1. Introduction

Soil organic matter (SOM) is the nub of ecosystem sustainability that plays a vital role in maintaining the soil fertility, structure, and stability. A substantial portion of SOM is stored in aggregates of variable size. The SOM is hypersensitive to the global climatic anomalies, specifically under the rising temperature scenario [1]. Rising temperature provides a congenial environment for microbes-mediated soil organic carbon (SOC) mineralization that result in significant loss in the sub-tropical climate [2,3]. Additionally, SOC mineralization is also regulated by the crop management practices such as nutrient/irrigation, tillage, cropping systems, and residue management [4]. The tillage-intensive rice (*Oryza sativa* L.)–wheat (*Triticum aestivum* L.) system in India has witnessed problems like loss of SOC stock, reduction in soil microbial biodiversity, and disintegration of soil aggregates [5,6]. Conservation tillage with crop residue retention has the potential to improve SOC stock and restore the soil health [7,8]. Theoretically, active SOC fraction like potentially mineralizable carbon (C_p_) is highly correlated with the crop management practices [9]. Conventional tillage exposes the protected SOC to the ambient temperature [10]. Global temperature is likely to increase by 0.3–0.7 °C in the next 15 years [11]. Therefore, it is imperative to comprehend the sensitivity of SOC and aggregate-associated SOC to the rising temperature under variable tillage systems and residue management [12].

Previous studies failed to show a consistent relationship between temperature and SOC mineralization under zero or no tillage with either increase [13,14,15] or decrease [16,17] or with no effect. The inconsistent results were due to duration of tillage and/or temporal environmental conditions. Therefore, experiments with temporal variation are needed to draw concrete evidence and unfold the underlying mechanism related to tillage practices and SOC decomposition or accumulation [18]. Six et al. [19] stated that experimental duration is one of the key factors to calculate the SOC mineralization. Zero tillage (ZT) would only be effective if it is implemented for more than 10 years. Crop residue along with tillage is also important for microbial diversity and SOC mineralization by supplying labile and moderately labile organic C [17]. The decomposed products are derived from residue decompositions along with soil particles form the macro-aggregates that protect SOC from further mineralization [19]. Thus, the interactive influence of tillage practices and residue management is very important and challenging, particularly in the long run.

Complex kinetics of SOM hinders the study of temperature sensitivity of SOM decomposition in the soil [20]. Temperature sensitivity (Q_10_) of SOM decomposition can be interpreted either by the exponential models or by Arrhenius models [20,21]. The exponential models give a clear representation of SOM decomposition. The Arrhenius model is rather mechanistic [22]. Complexity of organic compounds associated with a higher Arrhenius function indicates a proportional relationship between ‘recalcitrance’ of SOM and temperature sensitivity [20]. The difference in decomposition in the SOM is due to the activation energy (Ea) that delineates the minimum amount of energy needed to perform a specific reaction [20]. Higher Ea is required for a stable SOM and it would be affected minimally with the rise in global temperature [22]. Multiple factors like aggregate size distribution, microbial activity, nature of crop residue incorporated, extent of tillage, and experimental duration govern the nature of SOM sensitivity to the external temperature changes [23,24]. However, previous studies reported that conservation tillage along with sustainable residue management improved SOC content macro-aggregates by 67.1% and that the rest of the amount in micro-fractions was due to higher C-preservation capacity in the latter than the former [25]. The aggregate size fractions, such as macro-aggregates (250–2000 μm), micro-aggregates (250–530 μm), and silt-clay (<53 μm) also alter the SOM decomposition [26,27]. Biochemical quality of SOM, such as molecular weight, complexity, nature of chemical bond, and stability modify the temperature sensitivity of SOM. However, very few literatures are available on the impact of temperature sensitivity on different aggregate size and SOM under varied tillage and residue management practices in the rice–wheat system of the Indo-Gangetic plain (IGP).

Soil microbial diversity plays a pivotal function in the nutrient cycling, organic matter decomposition, and soil health which varies a lot under elevated temperature [28]. Enzymes like β-glucosidase and β-galactosidase are important for degrading the carbonaceous components in the soil and act as substrates for soil microbial functions [29]. Peroxidase and polyphenol oxidase are also vital for degrading the lignin and phenolic compounds in the soil [30], while glomalin is a glycoprotein produced by the arbuscular mycorrhizae that have a crucial role in the soil aggregation [31]. Previous studies strongly suggest the beneficial role of glomalin in soil C sequestration and in improving the soil’s physical condition [32,33]. However, information is meager on the glomalin production under different tillage and residue management practices in the Inceptisol.

Long-run CA with different tillage and residue management provides information on the impact of aggregate-associated carbon, carbon cycling, and soil microbial functions (i.e., enzymes activity). It could be highly useful in predicting the soil carbon mineralization/sequestration under the changing climate. We hypothesized that: (i) conservation agriculture significantly impacted temperature sensitivity and activation energy of SOM in the bulk and aggregated soil; (ii) ZT with added residues needs more activation energy than conventional tillage (CT) and residue burning. The objectives were: (i) to assess the impact of 18 years of CA on SOC mineralization and temperature sensitivity in an Inceptisol in the rice–wheat system and (ii) to determine the aggregate-associated C and enzymatic activity. 

## 2. Materials and Methods

### 2.1. Site Description

The soil samples for the present study were collected from a long-term (18 years old) conservation agriculture experiment at ICAR-Indian Institute of Farming System Research (ICAR-IIFSR), Meerut, Uttar Pradesh (28.99°N latitude; 77.70°E longitude). The climatic condition of the Meerut is hot summer with cool winters. The mean annual atmospheric temperature of Meerut is 24.1 °C with maximum and minimum temperatures of 39.1 °C and 7.2 °C, respectively. The mean annual precipitation is 840–880 mm. The taxonomic classification of the soil type is Inceptisol (Hyperthermic Ustept Haplustept). The basic soil properties are given in Supplementary Table S1.

### 2.2. Experiment Details and Crop Management

The conservation agriculture (CA) experiment was initiated during 1998–99 with the rice–wheat cropping system. The experiment consisted of three tillage and residue management practices with four replications in a split plot design. Tillage practices were kept in main plots that included: (i) zero tillage (ZT); (ii) conventional tillage (CT), and (iii) strip tillage (ST). Three residue management practices in sub-plots included: (i) residue burning (RB); (ii) no residue (NR), and (iii) 40% crop residue retention (R). Standard package of practices have been followed in both crops.

### 2.3. Soil sampling and Processing

After wheat harvest (end of March, 2016), soil samples were collected from two depths, namely, (i) 0–5 cm and (ii) 5–15 cm in triplicate manner, and later on, bulked and portioned into three sub-samples to make working samples. One portion of soil is grounded through a mortar, passed through an 8 mm sieve, and considered as bulk soil. The second portion of bulk soil is kept in the refrigerator (4 °C) for analyzing all microbial parameters. The third soil sample is pestle passed through an 8 mm sieve and utilized for analyzing aggregate size distribution. Sieved soil samples were used for analyzing all kinds of all other parameters, and visible dirt, plant materials, and gravels were removed while collecting soil samples from the field.

### 2.4. Aggregate-Associated C Analysis

Wet sieving technique and isotopic ratio mass spectrometer (IRMS) were conducted to differentiate soil samples into different aggregates and determine their total carbon (TC) content, respectively [34,35,36]. Total inorganic carbon (TIC) content was measured using the titrimetric method [37]. The total soil organic carbon (TSOC) content was calculated by subtracting the value of TIC from TC.

### 2.5. Carbon Mineralization Study

Carbon mineralization study was conducted in bulk (B), macro-aggregates (MA), and micro-aggregates (MI) in the 0–5 cm soil depth. A 25 g soil sample was taken for estimation of carbon mineralization. A triplicate set of soil samples were incubated in 250 mL conical flasks with alkali traps (10 mL of 0.5 N NaOH solution), and at each sampling depth while removing NaOH, compressed air was circulated and soil samples were maintained at 75% of field capacity (FC). The FC at −33 kPa of different soil samples were determined by using pressure plate apparatus. The rate of CO_2_ production of the soil samples was measured from day 1 till day 59 days different time intervals. The amount of evolved CO_2_ was determined back titrating 0.5 N NaOH by 0.5 M HCl at pH 8.3 in presence of barium chloride (BaCl_2_). Prior to study, all the soil samples were kept in an incubator for 15 days at 25 °C at 3/4 th field capacity [38].

It is very impoertant to take the readings at different day intervals to obtain a straight line [39]. The equation used for CO_2_ flux measurement was:

CO_2_–C evolved (mg kg^−1^) = (A − B) × N × 6 (1)

where A and B are the volume (ml) of HCl consumed for titrating 10 mL 0.5 M NaOH in control (flask without soil) and soil; N is the normality of HCl, and 6 is the equivalent weight of C. An exponential model by Sanford and Smith [40] was used to determine C loss with time:

C_t_ = C_o_ (1 − e^-Kct^) (2)

where C_o_ represents the initial SOC, and C_t_ is the pool of C mineralized at time t, with decay rate Kc.

Van’t Hoff factor (Q_10_) was calculated using the following formula by Janssens and Pilegaard [41]:

Q_10_ = {(Rate of C mineralization at 37 °C/Rate of C mineralization at 27 °C)} ^(10/T^_2_^-T^_1_^)^ (3)

Activation energy was calculated using the Arrhenius equation by Hamdi et al. [42]:

Ea = R* ln (Q_10_)/{(1/T_1_) − (1/T_2_)} (4)

where R = 8.314 j/mol; T_1_ and T_2_ are temperatures indicating the 10 °C temperature range (T_1_ = 27 °C, T_2_ = 37 °C).

### 2.6. Microbial Parameter Estimation

Easily extractable glomalin (EEG) can be determined by autocalving 1 g air-dried soil with 8 mL of 20 mM citrate solution adjusted in pH 7.0 at 12 °C for 30 min [43]. Soil microbial biomass carbon (SMBC) was determined by the chloroform fumigation extraction method from Jenkinson and Ladd [44] with an extraction efficiency of 0.45:

Microbial biomass carbon = (OC_F −_ OC_UF_)/K_EC_ (5)

where OC_F_ and OC_UF_ are organic carbon extracted from fumigated and non-fumigated soil, respectively (expressed on an oven-dry basis), and K_EC_ is the efficiency of extraction. A K_EC_ = 0.45 [45], i.e., by which we can extract only 45% of the microbial carbon.

β-D-glucosidase and β-D-galactcosidase activities were determined colorimetrically, following the procedure given by Eivazi and Tabatabai [46]. Polyphenol oxidase activity was determined calorimetrically at 525 nm by taking 0.2 M catechol as substrate [30]. Peroxidise activity was also determined calorimetrically at 450 nm by taking 100 mL clear soil filtrate and 3,3′5,5’-tetramethylbenzidine (TMB) as substrate [47].

### 2.7. Statistical Analysis

All the soil properties were statistically analyzed using Analysis of Variance (ANOVA) for split plot design using Duncan’s Multiple Range Test. Fisher’s Least Significant Difference (LSD) test was used as a post hoc mean separation test (*p* < 0.05) using IASRI (Indian Agricultural Statistics Research Institute) portal. All figures were drawn using Microsoft Office Excel (2010) of Microsoft, Redmond, Washington, USA.

## 3. Results

### 3.1. Aggregate-Associated Soil-C and Soil Microbial Biomass Carbon (SMBC)

In the 0–5 cm soil layer, total SOC within macro-aggregates was highest and lowest in ZT with added residues (ZT-R) (7.7 g kg^−1^) and CT without residues (CT-NR) (5.5 g kg^−1^), respectively. Implication of residue addition is highly significant on macro-aggregate-associated SOC after 18 years of experimentation. Plots under NR and RB had ~25 and 20% lower SOC concentration within macro-aggregates than R, respectively. The content of total SOC was lower in micro-aggregates than its counterpart whatsoever the treatment. The highest SOC content was found in ZT-R (4.8 g kg^−1^) and the lowest in CT-NR (4.3 g kg^−1^). Residue addition had ~7% and 10% higher total SOC content in the 0–5 cm and 5–15 cm depths, respectively, than NR (Table 1).

The range of SMBC was between 319.3–599.7 µg C g^−1^ in the 0–5 cm soil layer and ZT-R recorded highest with the lowest being in CT-RB. Plots under ST and ZT recorded with ~70% higher SMBC than CT. Similarly, R plots showed positive impact on SMBC than NR and RB as retention of stubbles increased SMBC value by 20% and 28.5 % in 0–5 cm depth and similar advantages can be visible in the subsequent depth (Table 1).

### 3.2. Soil Organic Carbon Mineralization

**Figure 1:**
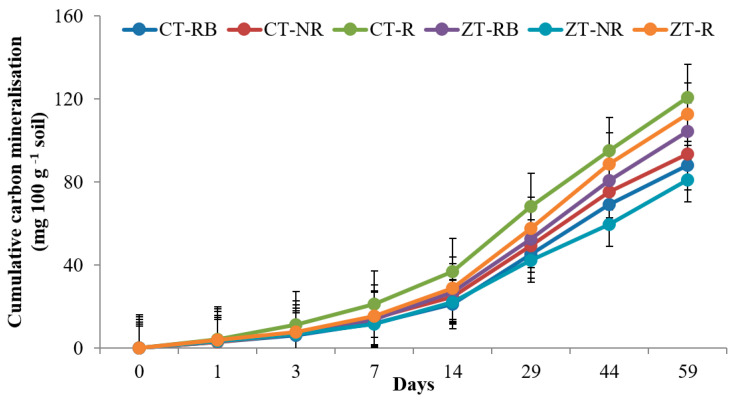
Cumulative carbon mineralization (mg 100 g^−1^ soil) of bulk soil in 0–5 cm depths as affected by 18 years of tillage and residue management in a rice–wheat cropping system in an Inceptisol at 37 °C. ZT = zero tillage; CT = conventional tillage; NR = no residue; RB = residue burning; R = residue retention. Error bars identify LSD (*p* ≤ 0.05) of different treatments.

**Figure 2:**
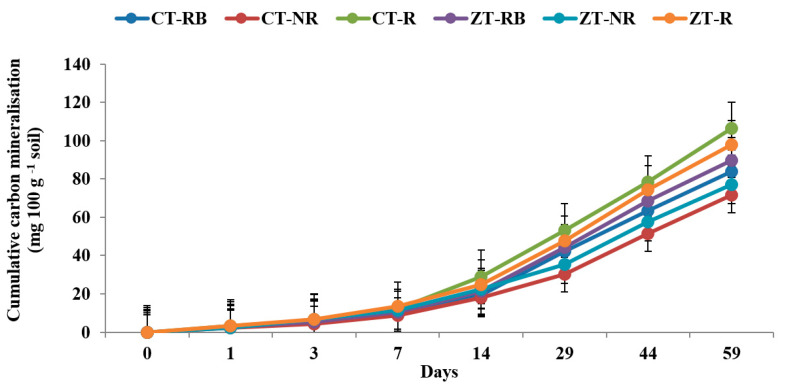
Cumulative carbon mineralization (mg 100 g^−1^ soil) of bulk soil in 0–5 cm depths as affected by 18 years of tillage and residue management in a rice–wheat cropping system in an Inceptisol at 27 °C. ZT = zero tillage; CT = conventional tillage; NR = no residue; RB = residue burning; R = residue retention. Error bars identify LSD (*p* ≤ 0.05) of different treatments.

The higher cumulative carbon mineralization (C_t_) from bulk soils was recorded in CT with added residues (CT-R) (4.12 and 120.5 mg 100 gm^−1^ on day 1 and 59, respectively) followed by ZT-R (3.98 and 112.7 mg 100 gm^−1^) in the 0–5 cm layer. Soil organic carbon (SOC) mineralization was ~48% lower in ZT-NR as compared to CT-R at the end of incubation. In the initial 7 days from the 0–5 cm layer, carbon mineralization was highest from ZT-R plots whereas after 7 days, maximum CO_2_ evolved from CT-R. At the end of 59 days, maximum Ct value was recorded in CT-R (106.2 mg 100 g^−1^) and the lowest was in CT-NR (71.5 mg 100 g^−1^) (Figure 1 and Figure 2).

**Figure 3:**
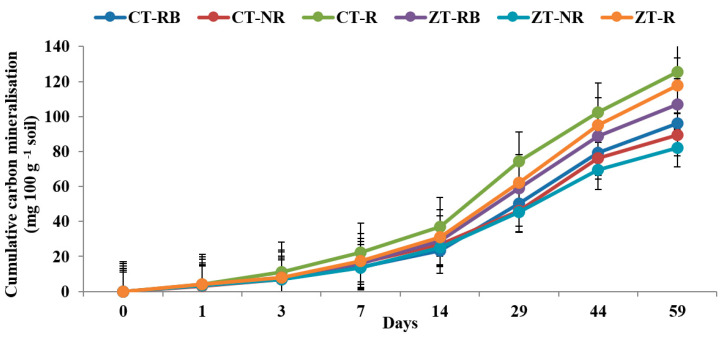
Cumulative carbon mineralization (mg 100 g^−1^ soil) from macro-aggregate in 0–5 cm depths as affected by 18 years of tillage and residue management in a rice–wheat cropping system in an Inceptisol at 37 °C. ZT = zero tillage; CT = conventional tillage; NR = no residue; RB = residue burning; R = residue retention. Error bars identify LSD (*p* ≤ 0.05) of different treatments.

**Figure 4:**
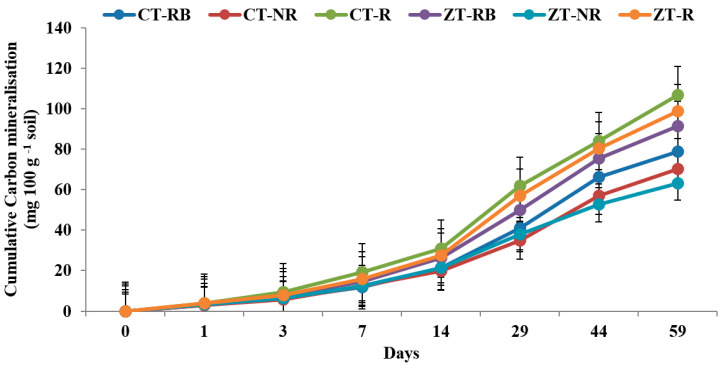
Cumulative carbon mineralization (mg 100 g^−1^ soil) from macro-aggregate in 0–5 cm depths as affected by 18 years of tillage and residue management in a rice–wheat cropping system in an Inceptisol at 27 °C. ZT = zero tillage; CT = conventional tillage; NR = no residue; RB = residue burning; R = residue retention. Error bars identify LSD (*p* ≤ 0.05) of different treatments.

**Figure 5:**
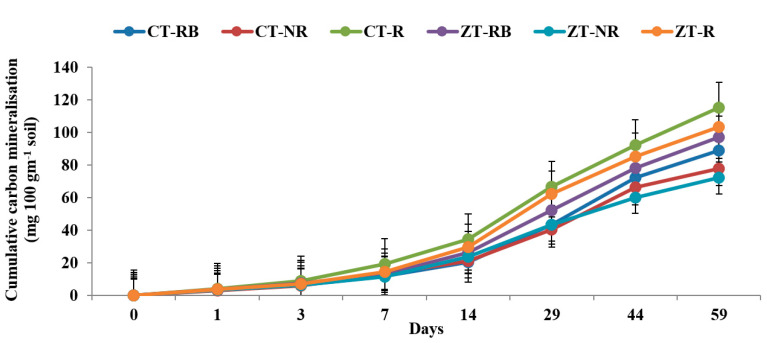
Cumulative carbon mineralization (mg 100 g^−1^ soil) from micro-aggregate in 0–5 cm depths as affected by 18 years of tillage and residue management in a rice–wheat cropping system in an Inceptisol at 37 °C. ZT = zero tillage; CT = conventional tillage; NR = no residue; RB = residue burning; R = residue retention. Error bars identify LSD (*p* ≤ 0.05) of different treatments.

**Figure 6:**
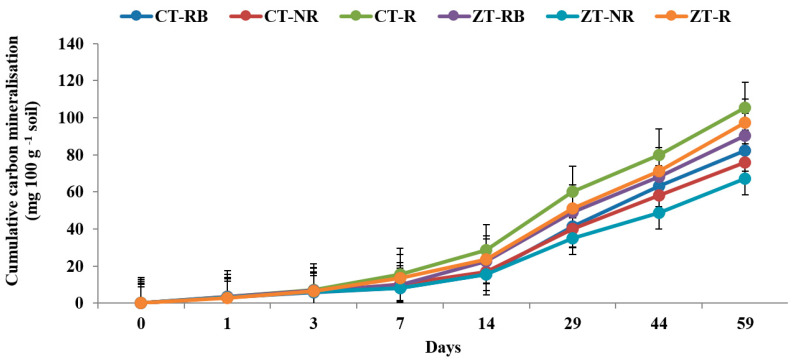
Cumulative carbon mineralization (mg 100 g^−1^ soil) from micro-aggregate in 0–5 cm depths as affected by 18 years of tillage and residue management in a rice–wheat cropping system in an Inceptisol at 27 °C. ZT = zero tillage; CT = conventional tillage; NR = no residue; RB = residue burning; R = residue retention. Error bars identify LSD (*p* ≤ 0.05) of different treatments.

In the case of macro-aggregates present in the top 5 cm, CT-R registered higher Ct value (125.5 mg 100 gm^−1^) which was 34.4% higher than ZT-R. Irrespective of temperature, residue-burned and residue-removed plots under CT recorded lower Ct value than the CT-R (Figure 3 and Figure 4). In the micro-aggregates, a similar trend was found, where CT-R plots registered highest Ct value over ZT-R in both the temperature. In 37 °C and 27 °C, the CT-R plots had ~10% and 7.6% higher Ct than ZT-R in the micro-aggregate fractions (Figure 5 and Figure 6).

### 3.3. Van’t-Hoff Factor (Q_10_) and Activation Energy (Ea)

The impact of tillage was non-significant for Van’t-Hoff factor (Q_10_) in bulk soils. Plots under ZT-R had higher Van’t-Hoff factor (1.18) than CT-RB plots (1.07). Added residues resulted in ~7% and 10% higher Q_10_ values than residue burning (RB) plots in macro- and micro-aggregates (Table 2). Activation energy (Ea) varied from 4.39–8.94 kJ mol^−1^ with the highest value in ZT-R and the lowest in CT-RB in the bulk soil (Table 2). Zero-tilled plots had significantly higher Ea value than the CT. Residue management followed the order of: residue addition (8.9 kJ mol^−1^) > no residue (8.34 kJ mol^−1^) > residue burning (5.14 kJ mol^−1^) in the bulk soil. The Ea in ZT was 25% and 18% higher in macro- and micro-aggregates than CT, respectively. Added residues had 44% and 60% higher Ea values than NR and RB, respectively (Table 2). The Ea and Q_10_ is significantly varied among different tillage and residue management practices. Irrespective of aggregate fraction, the Ea and Q_10_ value was significantly higher in zero-tilled and residue-retained plots. In cases of macro- and micro-aggregate ZT plots, they have 25.6% and 18.2% higher Ea values than CT plots, respectively. Similarly, Q_10_ value was also significantly higher in macro-aggregate fraction of ZT but in the case of micro-fractions, there was no significant difference. In case of residue management, both R and NR plots were found at par with respect to Ea and Q_10._ As compared to RB plots, the Ea value in macro- and micro-aggregate fraction was higher by ~45% and 60%, respectively, in residue-retained plots. The Q_10_ value was 7% and 10% higher in macro- and micro-aggregates in R plots as compared to RB plots in macro- and micro-aggregates, respectively. The interaction of tillage and residue management was found significant in the cases of both the fractions and thermodynamic parameters (Table 2).

### 3.4. Decay Rate Constant (Kc)

The decay rate constant (Kc) was significantly higher in CT than ZT in both the temperatures (37 °C and 27 °C) in bulk soil. The Kc value was 18.6% and 15.1% higher in no residue than added residues. The Kc value in the CT plots was 63% and 69% higher in 37 °C and 27 °C in micro-aggregates, respectively. Irrespective of temperature, residue management follows: R > RB > NR for Kc value (Table 3).

### 3.5. Glomalin Content

**Figure 7:**
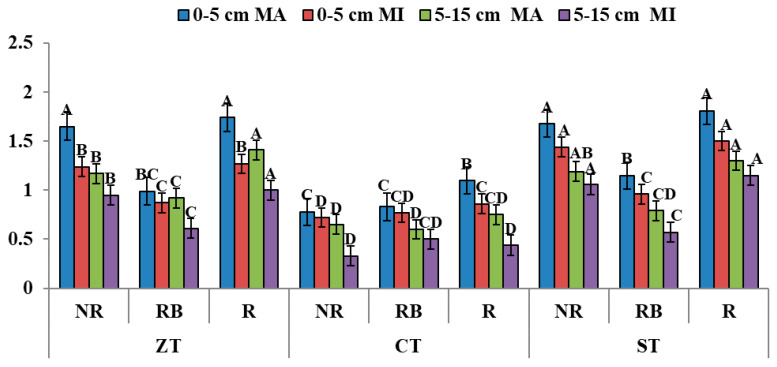
Glomalin content (mg g^−1^) of macro- and micro-aggregates in different depths as affected by 18 years of tillage and residue management in a rice–wheat cropping system in an Inceptisol. Different letters (A–D) for each parameter column show significant differences at *p* ≤ 0.05 by Duncan’s Multiple Range Test. ZT = zero tillage; CT = conventional tillage; ST = strip tillage; NR = no residue; RB = residue burning; R = residue retention; MA = Macro-aggregate; MI = Micro-aggregate. LSD value in 0–5 cm for MA: T: 0.16, RM: 0.15, T × RM: 0.25; for MI: T: 0.20, RM: 0.11, T × RM: 0.22; In 5–15 cm soil layer for MA: T: 0.14, RM: 0.13, T × RM: 0.22; for MI: T: 0.20, RM: 0.07, T × RM: 0.18.

Glomalin concentration within macro-aggregates was higher in ST than CT plots. Stubble burning had fatalistic impact on glomalin within macro-aggregates with 35 and 54% reduced concentration than residue removal and residue addition, respectively, in 0–5 cm (Figure 7). Irrespective of aggregate size, in the subsequent depth (5–15 cm), highest glomalin comtent was found in ZT-R plots (1.41 mg g^−1^) whereas lowest was in CT-NR (0.33 mg g^−1^). The defeatist impact of heat due to burning was evident in both the depths.

### 3.6. Enzymatic Activity

Extracellular enzymes like β- D-glucosidase activity ranged from 109.9–350.4 µg g^−1^ h^−1^ and 56.36–191.9 µg g^−1^ h^−1^ in 0–5 and 5–15 cm, respectively. Strip-tilled plots registered maximum β-D-glucosidase activity in the upper layer, contrary to the subsequent depth highest activity reported in the CT plots. Impact of residue burning was found highly detrimental for enzyme activity irrespective of tillage. In 0–5 cm and 5–15 cm, β- D-galactosidase content was highest in ZT-R plots (119.2 µg g^−1^ h^−1^) and ST-R plots (113.3 µg g^−1^ h^−1^) and the activity was higher by ~60% and ~84% as compared to CT-RB plots, respectively. In this case, the impact of heat was more prominent than β- D-glucosidase pointing to the cynical impact of RB on soil microbial function and diversity (Table 4). The trend of peroxidase activity was identical in both the depth and both the ZT and ST which were statistically at par. The activity of peroxidase in CT plots was ~50% lower in both depths in comparison with ZT. The impact of residue retention was properly visible in this case as both the NR and R plots showed almost the same peroxidase activity. However, RB had negative impact in this case, too (Table 5). Polyphenol oxidase is another recalcitrant C-degrading enzyme, found significantly higher in ZT and ST plots. In 0–5 cm and 5–15 cm, the activity of this enzyme was 31% and 13% higher in ST against CT. Like peroxidase, the impact of residue retention was not distinguished in the top layer (0–5 cm) but a noted difference can be visible in 5–15 cm as the trend follows: R > NR > RB. Except for the peroxidase activity in the 0–5 cm layer, the interaction of tillage and residue management was non-significant in the case of the recalcitrant C-degrading enzymes (Table 5).

## 4. Discussion

### 4.1. Aggregate-Associated Carbon and Soil Microbial Biomass Carbon

Zero tillage with residue addition significantly improved aggregate-associated C through continuous addition of organic matter via residues than the intensively tilled residue-burned plots. In plots of ZT under minimal disturbance together with protection from microbial degradation augmented aggregate-associated C. Lal et al. [48] reported aggregate stability and SOC content remain in hand-in-hand condition especially in the 0–5 cm layer. Bhattacharyya et al. [49] reported that nine years of conservation agriculture (CA) improved aggregate-associated C. They also highlighted the importance of slower decomposition rate along with the addition of root and stubble biomass. As per Lal et al. [48], C associated with the macro-aggregates had a higher stability over its counterpart (micro-aggregates) and the reasons were: (i) protracted decomposition rate with higher mean residence time; (ii) nominal physical disruption; (iii) continuous inclusion of surplus residue, and (iv) elevated microbial (fungal) activity which, in conjugation, boost up macro-aggregated C [48,50].

Although, in this instance, a non-significant effect was noticed between SOC and macro-aggregates, which might be due to a modest amount of clay present in the Inceptisol, clay played an indispensable role in soil aggregation along with soil minerals and protecting the SOM from further breakdown [51]. Stability from bio-chemical decomposition, anoxic conditions, formation of strong bond with solid states, limited microbial turnover, and formation of micro-aggregates inside macro-aggregates might be the reason behind the possible increase of macro-aggregate C than micro-aggregate C [52]. Soil microbial biomass carbon (SMBC) is an important indicator for soil quality and highly responsive to various management practices. Present study showed a positive impact of reduced tillage and residue retention on SMBC [53,54,55]. Retention of different crop residues induced availability of substrates rich in C and nutrients which resulted in a boom in microbial growth and biomass [56]. Moreover, physical disturbance due to intensive tillage and loss of valuable soil nutrients like nitrogen (N) and phosphorus (P) inhibited profuse microbial growth which resulted decrement in SMBC [57,58].

### 4.2. Carbon Mineralization

A higher C mineralization from the CT with added residue than ZT could be due to mechanical impedance in ZT [9,10]. Moreover, straw retention increased cumulative C mineralization due to the bio-chemical properties of the straw that potentially increased the level of labile C content in the soil [59,60,61]. Tillage disrupted soil structure and hastened the CO_2_ release from the C-rich macro-aggregate fractions [35]. Kan et al. [10] reported significantly higher C mineralization from the straw return plots [12,62,63]. Contradictions do exist, showing lesser C mineralization from the macro-aggregates than the micro-aggregates due to formation of particulate organic carbon in the soil [64]. The C:N ratio inside the aggregates is a vital indicator for C mineralization and higher C:N ratio is associated with higher aggregate class, therefore, elevated level of CO_2_ release formed the macro-fractions [65]. Less C mineralization in the micro-aggregates was due to lesser pore space that led to lesser space for the movement of water and nutrients and microbial activity [66,67]. The cumulative and potential C mineralization increased across treatments with residue additions. This may be due to an increase in SOC concentration with increase in straw input [68]. Retention of straw on the soil surface and subsequent ploughing increased C mineralization because of large-scale disruption of soil macro-aggregates and direct contact between micro-organisms and straws [69]. Reduced tillage and residue retention on soil surface prevented direct microbial contact and supplied a very less amount of nutrients to the microbes. Therefore, ZT along with residue retention was found to be an important option for protecting the SOC and curtailing the C mineralization.

### 4.3. Temperature Sensitivity (Q_10_), Activation Energy (Ea), and Decay Rate Constant (Kc)

Aggregate size is an important factor that significantly alters temperature sensitivity (Q_10_). The most common Q_10_ value used in soil organic matter (SOM) pools is 1.5–2 [20,42]. As per principle of thermodynamics, the decomposition rates of varying aggregate classes were more sensitive to the lower temperature than the higher one [70]. Higher temperature sensitivity in micro-aggregates than the bulk and macro-aggregates in ZT-R indicated micro-aggregates were sensitive to increasing temperature [71]. Wang et al. [22] reported a higher Q_10_ value in macro-aggregates. The differences in findings across regions could be due to the formation of the organo-mineral complex, bio-chemical nature of SOM, and edaphic conditions like soil moisture/temperature [72,73]. The reasons for higher Q_10_ value in micro-aggregates than the macro-aggregates were the formation of recalcitrant C in the macro-aggregates more than the micro-aggregates (Xie et al.) [74] and chemisorption mechanisms through ligand exchange and formation of polyvalent cation bridging [75]. The higher activation energy (Ea) in the ZT and residue-added plots indicated physical, chemical, and matrix-mediated protection due to residues which reduced with rise in temperature [76]. Clay encapsulation of the micro-aggregated SOC was protected from temperature stress and manifested higher sensitivity under elevated temperature conditions [77,78]. Decay rate constant of the micro-aggregate fractions were far higher than the macro-aggregates signifying C concentrations associated with the micro-aggregates were more temperature sensitive than the macro-aggregates.

### 4.4. Glomalin and Soil Enzyme Activity

Glomalin or glomalin-related soil protein (GRSP), a group of glycoprotein, has the ability to cement the soil particles together and form a better soil structure. The higher glomalin content in ZT is possibly because of higher fungal dominance or enhancement of fungal community composition [79]. Moreover, lesser physical disturbance and higher microbial activities increased glomalin content [80,81]. Previous study by Jaskulska et al. [82] reported that easily extractable glomalin content was highest under one pass-strip- tilled plots (OP-ST). The authors found that elevated glomalin content in OP-ST was correlated with higher aggregate stability. Additionally, intensive tillage led to disrupting the native arbuscular mycorrhizal fungi (AMF) community and glomalin content which is absent or minimal in ST plots [83]. The higher glomalin content in macro-aggregates than micro-aggregates is because of more labile C availability, presence of oxygen, and higher pore space which provides better opportunity to grow and proliferate [84]. Our results indicated the deleterious impact of residue burning on glomalin content, irrespective of tillage management. In lieu of the undeviating impacts of burning, high temperature disrupts the delicate host-microbe interaction and alters the bio-chemical properties of host plants which abate the glomalin production [85].

The leap of β-glucosidase activity in ST plots over ZT may be due to the congenial bio-chemical condition in the latter rather than the former, largely in the 0–5 cm layer, inducing higher proliferation and intensifying the decomposition of residues (C-rich material) [86]. Moreover, like glomalin, the impact of residue incineration is cataclysmic for the soil enzymes as it alters the microbial habitat by direct impact of oppressive heat [87]. Similarly, β-glucosidase plots under reduced tillage (both in strip and zero) exhibited inflated β-galactosidase activities in bulk soils. Unperturbed physical condition, sustained with diversified microbial growth and unabated C and nutrient supply in both ZT and ST, supervened higher enzyme activity [87]. Recalcitrant C-degrading enzymes (peroxidase and polyphenol oxidase) also followed the same path as the two earlier ones in the top layer (0–5 cm). Formation of recalcitrant C in the ZT due to protection from any physical or biological damage might have stimulated the microbes leading to higher enzyme content [88,89]. Formation of strong fungal network in the C-rich top soil and sequestration of C due to degradation of fungal cell wall components like melanin and chitin contributed to higher enzyme activity [90]. However, peculiarly, the higher polyphenol oxidase activity in CT plots could be due to the presence of oxygen which accentuated the SOC decomposition. Our results are in agreement with the previous studies by Chu et al. [90] and by Saikia et al. [91]. Hence, the impact of reduced tillage on soil microbial activity is well proved under long-term CA which becomes further amplified by residue retention.

## 5. Conclusions

Conservation agriculture for 18 years significantly influenced carbon mineralization. The activation energy of soil C mineralization was ~7% higher in micro-aggregate fractions than the macro-aggregates, irrespective of tillage management. Higher activation energy under ZT by ~6% and ~22% in bulk and aggregates than under CT is in accordance with our second hypothesis that confirms the potential of ZT plots to restore more carbon even in higher temperature. Higher Van’t-Hoff factor (Q_10_) in ZT-R plots, especially in the micro-aggregate fraction, implies sensitivity to micro-aggregates in higher temperature and protection of C in recalcitrant pools in macro ones. It indicated that the higher carbon sequestration potential under ZT with residue retention (ZT-R) over the intensively tilled residue-burned plots might be due to better stability, continuous addition of organic matter, and physical protection. After 18 years of CA, SOC stock increased by 4% in ZT over CT. Soil microbial biomass carbon, glomalin, and soil enzyme concentration were drastically reduced under CT and residue burning. Glomalin content in the ST-R plots increased by ~52% over CT-R due to better aggregate stability and greater AMF diversity. Therefore, it can be concluded that ZT with added residues can minimize the carbon mineralization with increasing temperature along with boosting up the soil biological activity and SOC stock. 

## Figures and Tables

**Table 1:** Aggregate-associated total soil organic carbon (SOC) (g kg^−1^) and soil microbial biomass carbon (SMBC) (μg kg^−1^ dry soil) in bulk and aggregates as influenced by 18 years of tillage and residue management in a rice–wheat cropping system in an Inceptisol.

	Total SOC (g kg^−1^)				SMBC (μg kg^−1^ Dry Soil)	
	**0–5 cm**		**5–15 cm**		**0–5 cm**	**5–15 cm**
	**Macro-Aggregate**	**Micro-Aggregate**	**Macro-Aggregate**	**Micro-Aggregate**	**Bulk Soil**	**Bulk Soil**
Tillage						
ZT ^#^	6.57 ^A^ *	4.56 ^A^	6.16 ^A^	4.56 ^A^	524 ^A^	291.7 ^A^
CT	6.17 ^A^	4.43 ^A^	5.90 ^A^	4.35 ^A^	370 ^B^	171.6 ^B^
ST	6.23 ^A^	4.43 ^A^	6.10 ^A^	4.43 ^A^	472.5 ^A^	293.7 ^A^
Residue management						
NR	7.43 ^A^	4.33 ^B^	5.33 ^B^	4.20 ^B^	434.5 ^A^	242.6 ^B^
RB	5.96 ^B^	4.43 ^A^	5.66 ^B^	4.46 ^A^	388.5 ^B^	179.8 ^C^
R	5.56 ^C^	4.70 ^A^	7.16 ^A^	4.68 ^A^	543.6 ^B^	334.1 ^A^
Tillage × Residue management						
ZT-NR	5.7 ^A^	4.4 ^AB^	5.5 ^A^	4.3 ^A^	494.2 ^A^	249 ^A^
ZT-RB	6.3 ^A^	4.5 ^A^	5.7 ^A^	4.6 ^A^	478.2 ^A^	208.6 ^A^
ZT-R	7.7 ^A^	4.8 ^A^	7.3 ^A^	4.8 ^A^	599.7 ^A^	417.6 ^A^
CT-NR	5.5 ^A^	4.3 ^BC^	5.1 ^A^	4.1 ^A^	327.6 ^A^	188.7 ^A^
CT-RB	5.8 ^A^	4.4 ^B^	5.6 ^A^	4.35 ^A^	319.3 ^A^	133.9 ^A^
CT-R	7.2 ^A^	4.6 ^A^	7 ^A^	4.6 ^A^	463.1 ^A^	190.7 ^A^
ST-NR	5.5 ^A^	4.3 ^BC^	5.4 ^A^	4.2 ^A^	481.6 ^A^	290 ^A^
ST-RB	5.8 ^A^	4.4 ^AB^	5.7 ^A^	4.45 ^A^	368 ^A^	197.1 ^A^
ST-R	7.4 ^A^	4.7 ^A^	7.2 ^A^	4.65 ^A^	567.9 ^A^	394 ^A^

^#^ ZT = zero tillage; CT = conventional tillage; ST = strip tillage; NR = no residue; RB = residue burning; R = residue retention. * Values are expressed as a mean of three replicates, and different letters (^A–C^) for each parameter column show significant differences at *p* ≤ 0.05 by Duncan’s Multiple Range Test.

**Table 2:** Activation energy (Ea) and Van’t-Hoff factor (Q_10_) of SOC mineralization in bulk and aggregate soils of 0–5 cm as influenced by 18 years of tillage and residue management under a rice–wheat cropping system in an Inceptisol.

	Activation energy (Ea) (kJ mol^−1^)			Van’t-Hoff factor (Q_10_)		
	**Bulk Soil**	**Macro-Aggregate**	**Micro-Aggregate**	**Bulk Soil**	**Macro-Aggregate**	**Micro-Aggregate**
Tillage						
ZT ^#^	7.68 ^A^	11.32 ^A^	11.63 ^A^	1.10 ^A^	1.15 ^A^	1.14 ^A^
CT	7.24 ^B^	8.42 ^B^	9.51 ^B^	1.09 ^A^	1.11 ^B^	1.13 ^A^
Residue management						
NR	8.34 ^B^	10.73 ^A^	11.41 ^A^	1.11 ^A^	1.14 ^A^	1.15 ^A^
RB	5.14 ^C^	6.68 ^B^	6.03 ^B^	1.06 ^B^	1.08 ^B^	1.07 ^B^
R	8.9 ^A^	12.06 ^A^	13.69 ^A^	1.12 ^A^	1.16 ^A^	1.19 ^A^
Tillage × Residue management						
ZT-NR	8.48 ^A^	13.30 ^A^	12.50 ^A^	1.12 ^A^	1.18 ^A^	1.17 ^B^
ZT-RB	5.90 ^C^	7.69 ^C^	6.18 ^B^	1.07 ^A^	1.10 ^C^	1.08 ^D^
ZT-R	8.94 ^A^	12.99 ^A^	15.03 ^A^	1.12 ^A^	1.18 ^A^	1.21 ^A^
CT-NR	8.21 ^B^	8.17 ^C^	10.33 ^A,B^	1.11 ^A^	1.11 ^C^	1.14 ^C^
CT-RB	4.39 ^D^	5.97 ^D^	5.89 ^B^	1.05 ^A^	1.07 ^D^	1.07 ^D^
CT-R	8.86 ^A^	11.14 ^B^	12.35 ^A^	1.12 ^A^	1.15 ^B^	1.17 ^B^

^#^ ZT = zero tillage; CT = conventional tillage; NR = no residue; RB = residue burning; R = residue retention. Values are expressed as a mean of three replicates, and different letters (^A–D^) for each parameter column show significant differences at *p* ≤ 0.05 by Duncan’s Multiple Range Test.

**Table 3:** Decay rate constant (Kc) of SOC mineralization in bulk and aggregate soils of 0–5 cm as influenced by 18 years of tillage and residue management under a rice–wheat cropping system in an Inceptisol.

	**Decay Rate Constant (Kc)** **(mg C per Week) (37 °C)**			**Decay Rate Constant (Kc)** **(mg C per Week) (27 °C)**		
	Bulk Soil	Macro-Aggregate	Micro-Aggregate	Bulk Soil	Macro-Aggregate	Micro-Aggregate
Tillage						
ZT ^#^	0.003853 ^B^	0.003595 ^A^	0.004513 ^B^	0.003487 ^B^	0.003112 ^A^	0.003927 ^B^
CT	0.005163 ^A^	0.003587 ^A^	0.004817 ^A^	0.004692 ^A^	0.003213 ^A^	0.004218 ^A^
Residue management						
NR	0.004923 ^A^	0.003430 ^B^	0.004080 ^C^	0.004420 ^A^	0.002984 ^C^	0.003575 ^C^
RB	0.004003 ^C^	0.003614 ^B^	0.004306 ^B^	0.003749 ^C^	0.003308 ^A^	0.003932 ^B^
R	0.004596 ^B^	0.003999 ^A^	0.005609 ^A^	0.004099 ^B^	0.003196 ^B^	0.004710 ^A^
Tillage × Residue management						
ZT-NR	0.003999 ^D^	0.003256 ^D^	0.004198 ^D^	0.003598 ^D^	0.002931 ^D^	0.003780 ^C^
ZT-RB	0.003625 ^E^	0.003443 ^C^	0.004093 ^DE^	0.003358 ^E^	0.003188 ^B^	0.003675 ^C^
ZT-R	0.003934 ^D^	0.003395 ^CD^	0.005248 ^B^	0.003506 ^D,E^	0.002872 ^D^	0.004328 ^B^
CT-NR	0.005848 ^A^	0.003604 ^B^	0.003962 ^E^	0.005243 ^A^	0.003037 ^C^	0.003373 ^D^
CT-RB	0.004382 ^C^	0.003785 ^B^	0.004519 ^C^	0.004141 ^C^	0.003248 ^B^	0.004189 ^B^
CT-R	0.005259 ^B^	0.004063 ^A^	0.005917 ^A^	0.004692 ^B^	0.003520 ^A^	0.005093 ^A^

^#^ ZT = zero tillage; CT = conventional tillage; NR = no residue; RB = residue burning; R = residue retention; NS = non-significant. Values are expressed as a mean of three replicates, and different letters (^A–E^) for each parameter column show significant differences at *p* ≤ 0.05 by Duncan’s Multiple Range Test.

**Table 4:** Soil β- D-glucosidase and β- D-galactosidase activities (µg g^−1^ h^−1^) as influenced by 18 years of tillage and residue management under a rice–wheat cropping system in an Inceptisol in 0–5 cm and 5–15 cm depth.

	β- D-Glucosidase		β- D-Galactosidase	
	**0–5 cm**	**5–15 cm**	**0–5 cm**	**5–15 cm**
Tillage				
ZT ^#^	230.5 ^B^	104.6 ^C^	88.53 ^A^	53.5 ^B^
CT	152.3 ^C^	167.8 ^A^	54.19 ^B^	38.39 ^C^
ST	248.2 ^A^	156.8 ^B^	84.57 ^A^	67.44 ^A^
Residue management				
NR	224 ^B^	139.3 ^B^	82.1 ^B^	32.76 ^B^
RB	132.2 ^C^	82.71 ^C^	47.8 ^C^	30.22 ^B^
R	274.9 ^A^	207.2 ^A^	97.33 ^A^	96.36 ^A^
Tillage × Residue management				
ZT-NR	271.1 ^B^	166.8 ^B^	93.82 ^B^	44.54 ^E^
ZT-RB	146 ^E^	104.8 ^D^	53.12 ^D^	17.60 ^G^
ZT-R	274.4 ^B^	191.9 ^A^	119.2 ^A^	98.37 ^B^
CT-NR	147.3 ^E^	84.16 ^E^	48.08 ^D^	18.53 ^G^
CT-RB	109.9 ^F^	56.36 ^F^	47.32 ^D^	19.30 ^G^
CT-R	199.9 ^D^	133.4 ^C^	67.17 ^C^	77.34 ^C^
ST-NR	253.7 ^C^	167.2 ^B^	105.1 ^B^	35.20 ^F^
ST-RB	140.6 ^E^	86.95 ^E^	42.97 ^D^	53.57 ^D^
ST-R	350.4 ^A^	176.2 ^B^	105.6 ^B^	113.37 ^A^

^#^ ZT = zero tillage; CT = conventional tillage; ST = strip tillage; NR = no residue; RB = residue burning; R = residue retention. Values are expressed as a mean of three replicates, and different letters (A–F) for each parameter column show significant differences at *p* ≤ 0.05 by Duncan’s Multiple Range Test.

**Table 5:** Soil peroxidase and polyphenol oxidase activities (µg g^−1^ h^−1^) as influenced by 18 years of tillage and residue management under a rice–wheat cropping system in an Inceptisol in 0–5 cm and 5–15 cm depth.

	Peroxidase		Polyphenol Oxidase	
	**0–5 cm**	**5–15 cm**	**0–5 cm**	**5–15 cm**
Tillage				
ZT ^#^	175.9 ^A^	118.3 ^A^	537.05 ^A^	202.2 ^C^
CT	89.93 ^B^	58 ^B^	365.16 ^B^	416.5 ^B^
ST	167.1 ^A^	115.3 ^A^	532.5 ^A^	479.3 ^A^
Residue management				
NR	160.4 ^A^	103.8 ^A^	566.47 ^A^	381.5 ^B^
RB	105.9 ^B^	77.51 ^B^	275.66 ^B^	239.3 ^C^
R	166 ^A^	110.4 ^A^	592.58 ^A^	477.3 ^A^
Tillage × Residue management				
ZT-NR	223.9 ^A^	129.6 ^A^	629.36 ^A^	151.2 ^A^
ZT-RB	115.7 ^B^	91.76 ^A^	337.48 ^A^	117.2 ^A^
ZT-R	188.6 ^A^	133.5 ^A^	644.32 ^A^	206.6 ^A^
CT-NR	94.91 ^B^	58.31 ^A^	451.57 ^A^	103.9 ^A^
CT-RB	68.04 ^C^	44.72 ^A^	171.89 ^A^	72.78 ^A^
CT-R	105.2 ^B^	70.97 ^A^	472.01 ^A^	184.9 ^A^
ST-NR	162.4 ^AB^	123.5 ^A^	618.47 ^A^	193.9 ^A^
ST-RB	133.6 ^B^	96.05 ^A^	317.60 ^A^	148.5 ^A^
ST-R	204.5 ^A^	126.4 ^A^	661.42 ^A^	227.6 ^A^

^#^ ZT = zero tillage; CT = conventional tillage; ST = strip tillage; NR = no residue; RB = residue burning; R = residue retention. Values are expressed as a mean of three replicates, and different letters (^A–C^) for each parameter column show significant differences at *p* ≤ 0.05 by Duncan’s Multiple Range Test.

## Data Availability

The data and materials are available from the corresponding authors upon reasonable request.

## Supplementary Materials

The following supporting information can be downloaded at: https://www.mdpi.com/article/10.3390/ijerph20010810/s1, Table S1: Initial soil properties (0–15 cm) of the experimental site
