# Supplementary material for: Conventional and Zero Tillage with Residue Management in Rice–Wheat System in the Indo-Gangetic Plains: Impact on Thermal Sensitivity of Soil Organic Carbon Respiration and Enzyme Activity
Source: Int J Environ Res Public Health. 2023 Jan 1;20(1):810. doi: 10.3390/ijerph20010810 (PMC9819925; doi:10.3390/ijerph20010810)
Supplement: Supplementary file 1 [file ijerph-20-00810-s001.zip › ijerph-2079816-supplementary.pdf]

Supplementary Table S1: Initial soil properties (0–15 cm) of the experimental site

**Variables**	**Value**
Texture	Sandy-loam
Bulk density (Mg m^{-3})	1.5
Soil pH (1:2.5 H_2O)	7.2
Organic carbon (g kg^{-1})	6.5
CEC # (Cmol kg^{-1})	39
Available N (kg ha^{-1})	112
Available P (kg ha^{-1})	3.9
Available K (kg ha^{-1})	102.5
DTPA $ extractable Zn (ppm)	0.25
DTPA extractable Cu (ppm)	0.05
DTPA extractable Fe (ppm)	0.6
DTPA extractable Mn (ppm)	0.4

#CEC = Cation exchange capacity; $DTPA = Di-ethylene triamine pentaacetate
